# Have we got the selection process right? The validity of selection tools for predicting academic performance in the first year of undergraduate medicine

**DOI:** 10.15694/mep.2017.000042

**Published:** 2017-03-09

**Authors:** Marita Lynagh, Brian Kelly, Graeme Horton, Ben Walker, David Powis, Miles Bore, Donald Munro, Ian Symonds, Graham Jones, Amanda Nagle, Tim Regan, Patrick McElduff, Michael David

**Affiliations:** 1 University of Newcastle; 2 University of New England

**Keywords:** Selection, admissions

## Abstract

This article was migrated. The article was marked as recommended.

**Content:** There remains much debate over the ‘best’ method for selecting students in to medicine. This study aimed to assess the predictive validity of four different selection tools with academic performance outcomes in first-year undergraduate medical students.

**Methods:** Regression analyses were conducted between admission scores on previous academic performance - the Australian Tertiary Admission Rank (ATAR), the Undergraduate Medicine and Health Sciences Admission Test (UMAT), Multiple-Mini Interview (MMI) and the Personal Qualities Assessment (PQA) with student performance in first-year assessments of Multiple Choice Questions, Short Answer Questions, Objective Structured Clinical Examinations (OSCE) and Problem-Based Learning (PBL) Tutor ratings in four cohorts of students (N = 604, 90%).

**Results:** All four selection tools were found to have significant predictive associations with one or more measures of student performance in Year One of undergraduate medicine. UMAT, ATAR and MMI scores consistently predicted first year performance on a number of outcomes. ATAR was the only selection tool to predict the likelihood of making satisfactory progress overall.

**Conclusions:** All four selection tools play a contributing role in predicting academic performance in first year medical students. Further research is required to assess the validity of selection tools in predicting performance in the later years of medicine.

## Introduction

The most appropriate methods for selecting undergraduate medical students continues to generate debate in Australia ( Wilson 2012) and overseas ( Ferguson 2002). Medical graduates are expected to possess a range of personal skills and traits beyond medical knowledge and clinical skills. Thus discussion has tended to focus on determining how best to assess applicants’ cognitive and personal attributes in a manner that is both transparent and equitable ( Adam et al. 2012). Although previous academic performance scores have been shown to be strong predictors of medical student performance ( Ferguson 2002; Wilkinson et al. 2011; Shulruf et al. 2012), they do not provide a holistic picture of an applicant’s ability to achieve success in a medical degree and enter the medical profession ( Doherty & Nugent 2011). Additionally, relying solely on prior academic performance can reduce opportunities of applicants from low socio-economic backgrounds ( Griffin & Hu 2015) and limits the ability to discriminate between high academic achieving applicants ( Wilkinson et al. 2011).

In order to increase transparency in the selection process and reduce potential sources of bias, many university medical programs now utilise a range of selection tools. These include interview panels or multiple mini-interviews (MMIs) ( Eva et al. 2004), personality questionnaires (e.g., the Personal Qualities Assessment [PQA]) ( Powis et al. 2005) and verbal and non-verbal logic tests (e.g., Undergraduate Medicine and Health Sciences Admission Test, UMAT). The implementation of these types of selection processes imposes significant time and resources costs for both medical schools and applicants, yet robust and consistent evidence regarding the validity of these selection tools in predicting first-year medical undergraduate performance is limited. There is a vital need to for more research on the predictive validity of these selection tools to ensure evidence-based, time- and cost-effective identification of appropriate students into undergraduate medical degrees.

### The University of Newcastle and University of New England Joint Medical Program Selection Process

**Figure 1.**
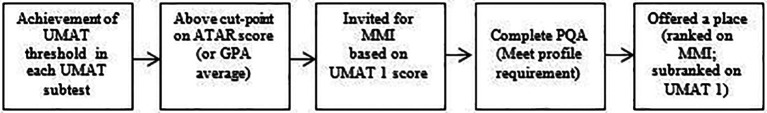
Selection process for students entering the Joint Medical Program (JMP)

In an effort to overcome barriers relating to the rural medical workforce shortage, a strategic partnership was entered into by the University of Newcastle and the University of New England (both located in New South Wales, Australia) and two local area health districts (Hunter New England; Central Coast) to deliver a Joint Medical Program (JMP) tailored to the current and projected needs of the region. A revised selection process for the JMP was initiated in 2012 utilising a combination of tools (see Figure 1). These include the Undergraduate Medicine and Health Sciences Admissions Test (UMAT); academic performance scores such as the Australian Tertiary Admission Rank (ATAR) or university grade point average (GPA); Multiple Mini-Interview (MMI) ( Eva et al. 2004); and the Personal Qualities Assessment (PQA) ( Powis et al. 2005).

### Current evidence for the predictive validity of selection tools

#### Undergraduate Health Science and Medical Admissions Test (UMAT)

The UMAT is an ‘aptitude’ test comprising three sub-tests: i) logical reasoning and problem solving (UMAT1); ii) emotional intelligence and empathy (UMAT2); and iii) non-verbal reasoning (UMAT3) ( Australian Council for Educational Research 2015). Studies exploring the predictive validity of the UMAT for first-year medical performance have typically reported weak to moderate relationships between the test and academic outcomes ( Wilkinson et al. 2011; Mercer & Puddey 2011; Shulruf et al. 2012; Edwards et al. 2013; Simpson et al. 2014). Wilkinson et al (2011) reported weak correlation coefficients of between 0.06-0.15 for total UMAT and each UMAT subtest with first-year GPA. Shulruff and colleagues (2012) reported significant linear regression coefficients ≤ 0.03 for UMAT1-3 against second, third, fourth year GPA scores, and Mercer and Puddey (2011) found significant but weak linear regression coefficients < .08 for UMAT1 against clinical skills. In contrast, Simpson and colleagues (2014) were unable to establish the predictive validity of UMAT against participants’ weighted average mark at any point across their six-year medical degree and Edwards et al (2013) reported mixed results regarding the relationship between UMAT and academic performance when comparing the results of three Australian universities. One medical school reported moderate correlation coefficients of between 0.40-0.48 between UMAT1 and GPA in three different year-cohorts. On the other hand, correlations between UMAT1 and GPA at the two other universities were notably weaker (0.03-0.18; 0.16-0.26) ( Edwards et al. 2013). The UMAT has been a mandatory selection tool for all medical schools in Australia with undergraduate entry programs since 1999.

#### Prior academic achievement

A meta-analysis by Ferguson et al (2002) suggested that when compared to personality tests and demographic characteristics, prior academic performance is the strongest predictor of undergraduate medical performance. This is supported by Mercer and Puddey (2011) who reported significant linear regression coefficients of up to 0.35. Similarly, Wilkinson and colleagues (2011) and Shulruff et al (2012) reported that prior academic achievement was the strongest predictor of academic performance in years one to four although the strength of this relationship tended to diminish over time. In contrast, Adam et al (2012) found that prior academic performance was the best predictor of year four and five performance. Together these findings suggest that prior academic performance is the strongest predictor of future academic performance across the entire medical undergraduate degree trajectory.

#### Interviews and Multiple Mini-Interviews (MMI)

Structured interviews have been considered an important element of the selection process for entry in to medicine for many years. Studies have demonstrated that interviews can predict higher scores in clinical assessment items ( Mercer & Puddey 2011) and total examination scores ( Wilkinson et al. 2008). Scores on structured interviews have also been shown to predict overall weighted average marks in addition to performance in specialty areas such as general practice unit scores and obstetrics and gynaecology unit scores ( Puddey & Mercer 2014). The Multiple Mini-Interview (MMI), recently adopted by many medical schools, is completed by candidates sequentially attending a series of stations designed to assess non-cognitive skills such as organisation, ethical sensitivity, and demystifying information ( Eva et al. 2004). Among a sample of Canadian undergraduates, the MMI was shown to be a strong predictor of Objective Structured Clinical Examination (OSCE) performance ( Reiter et al. 2007). A study in the UK similarly found that the MMI was a significant predictor of performance in an OSCE-based licensing examination in both undergraduate and postgraduate medical cohorts ( Eva et al. 2009) while another UK study reported that the MMI was the most consistent predictor of success in both written and OSCE-based assessments in the early years of medicine ( Husbands & Dowell 2013). The predictive validity of the MMI with performance in an Australian medical undergraduate program remains untested with a recent systematic review calling for further research in different cultural contexts ( Pau et al. 2013).

The MMI is essentially a structure for assessment of skills (with multiple brief interactions or tasks with standardised scoring), and studies of predictive validity will naturally be influenced by the specific skills assessed, the structure of the stations and their scoring characteristics. Hence the MMI is not a single entity or universally standardised test. Within the Joint Medical Program, the MMI-like assessment is referred to as “Multiple Skills Assessment” (MSA) in order to make explicit that it involves evaluation of a range of non-cognitive skills through structured tasks, as distinct from an interview-based assessment.

#### Personal Qualities Assessment (PQA)

Personality tests are increasing being used as a means of complementing medical student selection processes ( Wilson et al. 2012). They are often used to gain a more holistic understanding of an applicant, particularly their ability to cope with the stressors of a medical training program ( Tyssen et al. 2007; Horsburgh et al. 2009). However, the relationship between applicant personality and academic performance is unclear. For example, some studies have shown that traditional measures of personality, such as Costa and McRae’s (1992) ‘Big Five Model’ of personality, have shown no correlation with academic (GPA) or MMI performance ( Kulasegaram et al. 2010). In contrast, a review by Doherty and Nugent (2011) found that ‘conscientiousness’ was a strong positive predictor of academic performance over a seven-year period. The Personal Qualities Assessment (PQA) tool identifies applicants possessing undesirable personality characteristics and assists in discriminating between applicants with equally high academic performance ( Powis et al. 2005). PQA has been shown to predict clinical performance in years four and five, but not written exam performance ( Adam et al. 2015). Dowell and colleagues (2011) found no relationship between PQA and academic performance.

The selection process used by the University of Newcastle / University of New England JMP is unique in its combination of selection tools. Despite the UMAT being developed by the University of Newcastle in 1991 there has been limited research to determine the predictive validity of the UMAT alone, or in combination with other selection tools. More research is needed to validate individual selection tools and the combination of selection tools. The aims of this study were to:

1. Determine the individual predictive validity of four different selection tools (UMAT subtests; ATAR scores, MSA, PQA) with academic performance outcomes in first-year undergraduate medical students.

Determine the combined predictive validity of all four selection tools with academic performance outcomes in first-year undergraduate medical students.

## Methods

### Sample

All first-year undergraduate students from the 2011 to 2014 entry cohorts of the University of Newcastle and University of New England JMP were invited to participate in the study. Ethics approval was obtained from the Human Research Ethics committees at both Universities. Initial invitations were made during lectures with a reminder email sent two weeks later. Only data from students who returned signed consent forms indicating their willingness to participate were included in the study.

### Selection tools

Students’ ATAR, UMAT, MSA and PQA scores were all retrieved from secure databases at the University of Newcastle and the University of New England.

#### ATAR

The ATAR score is a student’s ranking relative to his or her peers upon completion of secondary school education. Scores are percentiles ranging from ‘less than 30’ up to a maximum of 99.95 (in increments of 0.05) and are routinely provided to universities centrally by The Universities Admissions Centre (UAC). The ‘cut-off’ or minimum required score to medical school varies each year and between universities, but is generally greater than 90.

#### UMAT

The three UMAT sub-tests (UMAT1 - *Logical Reasoning*; UMAT2 - *Understanding People;* UMAT3 - *Non-verbal Reasoning*) involve multiple-choice responses and must be completed within 4.5 hours. Raw scores for each sub-test are standardised to a mean of 50.0 and a standard deviation of 10.0. Applicants to the JMP are required to reach a threshold of 50.0 in each UMAT sub-test.

#### MSA

For students in the 2011 entry cohort, the selection process still utilised a 45 minute, 2-person structured panel interview. Interview scores for the 2011 cohort were retrieved and analysed separately. From 2012 onwards, the interview was replaced with a Multiple Skills Assessment (MSA, based on the MMI format). Applicants who meet UMAT and ATAR thresholds are invited to undertake a MSA at the university campuses in November/December. The MSA includes eight (8) stations designed to assess nine (9) different skills including: motivation, decision making, verbal communication, personal insight, teamwork, critical thinking, demystifying complex text, organisation skills, and ethical sensitivity. Verbal communication is scored based on independent assessment across a number of stations requiring verbal interaction, hence the 8 stations assess a total of 9 attributes. These domains were determined after consultation with key academic staff and community members. Students complete each 8-minute station with 2-minute intervals over a period of one hour.

#### PQA

On the same day of attending for their MSA, applicants complete an on-line assessment of non-cognitive personal qualities, the PQA ( www.pqa.net.au), under examination conditions. The PQA has been included in the JMP selection process since 2012. The PQA comprises three subtests:- the Narcissism, Aloofness, Confidence, Empathy (NACE) scale; the Interpersonal Values Questionnaire (IVQ); and the Self-Appraisal Inventory (SAI) - providing scores on four personal qualities:- moral orientation, involvement (vs detachment), self-control (vs disorderliness) and emotional resilience. A more detailed description of the subtests and qualities assessed has previously been reported ( Adam et al. 2012). The subtests of the PQA have previously demonstrated good internal consistency and construct validity: NACE α=0.9 ( Munro et al. 2005); IVQ α=0.83-0.92 ( Bore et al. 2005); SAI α > .80 (Bore et al. 2011).

### Outcome Measures

The primary outcome was students’ first-year academic performance as measured by summative in-course assessments using 1) multiple-choice exam questions (MCQs); 2) short-answer exam questions (SAQs); and 3) Objective Structured Clinical Examination (OSCE) scores. Students are assessed across three themes in the first year of their program: *Professional Practice, Medical Science* and *Public Health.* MCQs and SAQs largely assess knowledge and analytical skills across the three themes, while the OSCEs typically involve two stations that assess practical clinical skills, and interpersonal and communication skills with simulated patients. Student professional behaviour related to attendance, participation and engagement in tutorials was assessed using Problem Based Learning (PBL) Tutor rating scores. A secondary outcome measure of ‘satisfactory progress’ was defined as having no recorded ‘Not Satisfactory’, ‘Borderline’ or ‘Fail’ for any assessment item, or sub-component of an assessment item or PBL performance during the first year of studies.

### Data analysis

All student data were de-identified once linkage was completed. Pearson correlations were calculated between selection tool scores. Correlations were not corrected for range restriction. Both unadjusted and adjusted linear regression analyses were conducted for associations between selection tool scores and outcome measures of MCQs, SAQs, OSCE and PBL ratings. Logistic regression analyses were conducted to compare satisfactory versus unsatisfactory progress. A series of linear regression models were conducted to test different combinations of selection tools and predictive relationships with outcome measures. Variables with a p-value of ≤ 0.02 were included in the final regression analyses with a backwards-stepwise method used to exclude variables from the models. The final regression models used two-sided tests with a significance level of 5%. Statistical analyses were performed using STATA software, version 8.0.

## Results

### Study Sample

A total of 604 students consented to participate in the study, a response rate of 90%. The majority of students (67%) were enrolled at the Newcastle campus, were school leavers (65%), Australian citizens (88%) and did not identify as Aboriginal or Torres Strait Islander (95%). **Table 1** shows that, overall, the demographic characteristics were stable across each of the four cohorts with the exception of the 2014 cohort, in which significantly more males than females, and more non-school leavers than school-leavers were enrolled in the JMP compared to previous cohorts.

### Multiple Choice Question (MCQ) and Short-Answer Question (SAQ) performance

Table 2 shows outcomes of the linear regression analyses of MCQ and SAQ performance against demographic characteristics and selection scores, adjusted for age, sex, citizenship and school leaver status. Better MCQ performance was predicted by being a school leaver (p=0.008), male (p=0.029), older age (p=0.032), greater ATAR (p<0.0001), UMAT1 (p=0.047), UMAT2 (p=0.033) and MSA Total (p=0.012) scores. Individual MSA stations of Demystifying Text (p=0.002), Verbal Communication (p=0.034) and Ethical Sensitivity (p=0.032) were also significant predictors. Higher scores on SAQs were predicted by higher ATAR (p<0.0001), UMAT1 (p=0.018), UMAT3 (p=0.02) and individual MSA stations Demystifying Text (p=0.003) and Verbal Communication (p=0.044), and Self Control as measured in the PQA ( *p* = 0.047).

### Objective Structured Clinical Examination (OSCE) scores

Results of adjusted linear regression analyses of OSCE scores with demographic characteristics and selection tool scores are shown in **Table 3.** Among the selection tools, higher ATAR ( *p* < 0.001), UMAT2 (p = 0.014), PQA Self Control (p = 0.009) and individual MSA stations of Verbal Communication (p = 0.033) and Critical Thinking (p = 0.012) all predicted better OSCE performance.

### Problem Based Learning (PBL) Tutor Ratings

In regard to PBL Tutor ratings, being female (p = 0.005), older (p=0.006) and not being an Australian citizen (p = 0.02) were demographic characteristics that predicted higher PBL ratings. UMAT3 (p = 0.0005) and individual MSA stations of Decision Making (p = 0.043), Organisational Skills ( *p* < 0.0001) significantly predicted higher ratings. MSA stations of Verbal Communication (p = 0.001), Critical Thinking (p = 0.002) and Ethical sensitivity (p = 0.001) all predicted lower PBL Tutor ratings. (see **Table 3**)

### Predictors of satisfactory versus unsatisfactory progress

Table 4 shows the predictors of satisfactory versus unsatisfactory progress in the first year of the JMP. After adjusting for age, sex, citizenship and school leaver status, higher ATAR scores ( *p* = 0.0006) was the only selection tool that significantly predicted being more likely to make satisfactory progress through the first year of medicine.

### Combined Predictive Validity

Results of a series of linear regression models demonstrate that the combination of all four selection tools explained the most variation (R ^2^=12.8%) in MCQ scores in Year One, with ATAR scores by itself explaining 9.4% of variation. The model including all four selection tools explained 18% of variation in Year One SAQ scores and around 7.8% of variation in Year One OSCE scores.

## Discussion

This study aimed to assess the predictive validity of four selection tools with first year academic performance across domains that included knowledge acquisition, application of knowledge and aspects of professionalism and behavioural conduct. In doing so, the study aimed to establish whether the selection process for entry into the Joint Medical Program (JMP) is sound and appropriate. All of the current selection tools (i.e., ATAR, UMAT, MSA and PQA) were found to have significant predictive associations with one or more measures of student performance in Year One of an undergraduate medical program. Three of the four selection tools, UMAT, ATAR and MSA scores were consistently associated with first year performance on a number of outcomes. ATAR in particular had strong associations across all measures of first year performance with the exception of PBL Tutor ratings. ATAR was the only selection tool to predict the likelihood of making satisfactory progress overall compared to making unsatisfactory progress in Year One. This is consistent with a number of previous studies ( Ferguson et al. 2002; Wilkinson et al. 2008; Mercer & Puddey 2011; Shulruf et al. 2012; Adam et al. 2015) reporting that ATAR scores significantly predict student performance in medicine and more broadly with international literature on the predictive validity of prior academic achievement and medical school performance ( Poole et al. 2012). In the main this finding may be viewed as strong endorsement for the inclusion of prior academic achievement in the medical selection process. However, given evidence that ATAR scores have decreasing predictive capacity over program years ( Mercer & Puddey 2011; Poole et al. 2012) and prior academic results are socio-economically biased ( Mercer 2009; Cleland et al. 2012; Griffin & Hu 2015), their inclusion should be carefully balanced with assessment of other attributes so that their embedded inequity is countered with appropriate redress as part of the selection process.

Each of the three UMAT subtests were found to predict different outcome measures of Year One performance. Higher UMAT3 ( *Non-verbal Reasoning*) scores predicted better performance on SAQs and PBL Tutor ratings. This finding is somewhat surprising given previous studies have reported that UMAT3 has no predictive ability in the first year of study ( Wilkinson et al. 2011; Shulruf et al. 2012; Poole et al. 2012) and contrasts with other more recent evidence that UMAT3 is a significant negative predictor of clinical performance in Years 1-3 ( Mercer & Puddey 2011) and overall course performance ( Mercer 2009). This divergence in findings may be explained by differences in criterion measures used to assess student performance and course curricula. The present study attempted to test predictive ability against more course specific outcomes as opposed to overall GPA or course performance measures.

UMAT1 ( *Logical Reasoning*) scores significantly predicted higher first year MCQ and SAQ scores, consistent with the findings of Wilkinson and colleagues (2011). UMAT2 *(Understanding People)* scores predicted higher MCQ and OSCE scores in Year One. The findings in regard to both of these UMAT subtests appear coherent with the constructs they measure and attributes assessed in the outcome measures. For instance, one would expect that students who perform better in UMAT2 (Understanding People) would score higher in interactional and communication skills as assessed by OSCEs. Only one other recent study has provided evidence supporting the predictive ability of UMAT2 with student performance across program years (Hay et al. 2015). The lack of evidence to-date for the predictive validity of UMAT2 may be due to the disparity between the emotional intelligence constructs measured in this selection test and cognitive skills more traditionally evaluated in medical school assessments. Though the present study provides evidence for the predictive ability of each of the UMAT tests with performance in first year medicine, most of the associations are weak. Others have suggested that the predictive power of UMAT increases in the later years of a program ( Poole et al. 2012), so longer term validity studies are required to test these forecasts.

Six of the nine non-cognitive skills and/or attributes assessed in the individual MSA stations were found to be predictive of one or more first year student performance measures, though again many of the associations were weak. Five of these were predictive of PBL Tutor ratings, with organisational and verbal communication skills having the strongest predictive power. In addition to weaker associations with the attributes of ethical sensitivity, critical thinking and decision making, these findings are not unexpected given that these are the very skills required for students to succeed and engage in a problem-based learning curriculum, and further support the appropriateness of their inclusion in the selection process for the JMP program. Verbal communication skills were also (weakly) predictive of performance in MCQs, SAQs and OSCEs. This may be viewed as support for the importance of verbal communication skills both in the performance of medical students (and practitioners) and the need to appropriately screen for this proficiency as part of the selection process. Three of the MSA stations - motivation, personal insight and teamwork - were not associated with any outcome measure suggesting that either the student performance measures utilised did not adequately reflect these skills or that their inclusion in the selection process should be reconsidered in the future.

There are few previous studies with which to compare the current findings. A recent systematic review acknowledged the limited evidence for the predictive validity of MMIs ( Pau et al. 2013). This is the first study to explore the predictive validity of an MMI-based selection tool among a sample of Australian medical students. While the current findings appear promising and the existence of some evidence from Canada supporting the validity of MMIs in predicting performance in medical licensing examinations ( Poropat 2009; Pitt et al. 2014), further studies are needed to examine the predictive capacity of MMIs given their increasing use as a selection tool in many medical schools. Additionally, no existing literature has yet given consideration to ‘unpacking’ the structure, content, attributes and processes involved in the varying models of MMIs currently in use. The MMI is not one standardised assessment tool, but rather its predictive validity must depend on the qualities and skills evaluated and the processes for how these are assessed. Though the present study contained only one cohort of students for which an interview was used as part of the (old) selection process, it is interesting to note that the data showed no predictive ability of the interview with any measure of student performance in first year. This finding together with evidence that MMIs are not subject to SES-related bias ( Griffin & Hu 2015) provides vindication of the decision to replace the interview with an MMI-based tool in the JMP selection process in 2012.

Self-Control was the only sub-scale of the PQA to be predictive of any Year one outcome measure. Students with higher levels of self-control or conscientiousness were more likely to perform better at SAQs and OSCEs. This is consistent with the findings of Adam and colleagues (2012) who found that higher PQA Self Control scores significantly predicted higher OSCE scores and tutor assessments in Years 1 and 2 of medical studies in the UK. The association between conscientiousness and academic performance has been well supported in other educational research ( Poropat 2009; Pitt et al. 2014), though recent evidence by Adam et al (2015) suggests the predictive ability of this personal quality may diminish by the later years of study in medicine. Our results indicated no predictive relationships between the other non-cognitive variables of moral orientation, emotional resilience and involvement with first year student performance in contrast to other studies that have reported significant associations of these qualities with academic performance, professional behaviour and progression in medicine and nursing ( Adam et al. 2012; Pitt et al. 2014; Adam et al. 2015). The lack of associations between these qualities and student performance may in part be explained by differences in the selection of outcome measures used in the present study compared to previous research. A more feasible explanation may lie with the outcome measures themselves. There is incongruity between the non-cognitive skills, qualities or behaviours that personal qualities measures such as the PQA are believed to predict and the performance outcomes assessed by traditional, cognitive academic assessments (eg. MCQs, SAQs) ( Adam et al. 2012). If these qualities are viewed as important for success as a medical student and practitioner, then perhaps medical schools need to consider assessment of non-cognitive skills not only at entry but instruction and formal assessment throughout medical school education. In addition, it is important to note that unlike the other selection tools used for entry into the JMP, the PQA serves as a filter to select applicants who meet a particular profile of qualities considered suitable for success in a medical course. Thus the sample of students in the present study had already satisfied this profile. Further the profiling method utilised in the JMP is not a linear function of subscale scores, so significant correlations with performance scores are not expected. Establishing the predictive ability of the PQA for conventional medical school performance may not be an imperative, given that those applicants who are successful in being admitted to medicine have already established that they have suitable personality characteristics.

A second aim of this study was to determine the combined predictive validity of all four selection tools with performance in first-year undergraduate medicine. Results indicated that the current selection process used for the JMP program accounts for only approximately 13% of the variance in MCQ scores, 18% of SAQs and 8% of OSCE scores. While at first glance these values appear disappointing, they are comparable with the findings of Mercer and Puddey (2011) who reported that the combination of prior academic results, UMAT and interview scores explained 20% of the variance of knowledge-based assessments in Years One and Two but only 10% of clinical performance in the later years. Similarly, Poole et al (2012) reported that the predictive power for UMAT and admission GPA combined varied from 35% in Year Two GPA to only 8% in Year Five Clinical grades suggesting that the combined predictive validity of the selection tools may diminish over the years of a course. Some see this as contrary to expectations that selection tools should be better predictors of performance in the later years of medicine when assessments more closely reflect the skills of a medical practitioner ( Shulruf et al. 2012). An opposing view is that the predictive power of selection tools should be greatest in the early years of study as the selection process is focussed on identifying who will succeed as a medical student, not who will make a good clinician.

### Strengths and Limitations

There are a number of limitations with the present study. First, the choice of outcome measures used as criteria for student performance was constrained by the existing assessment methods and data records used in the first year of the JMP program. These are predominantly cognitive assessments combined with a number of ungraded ‘hurdles’ in which students must achieve a ‘Satisfactory’ result to progress. There are fewer systematic, formal assessments of non-cognitive skills and/or professional behaviour. Second, the study was confined to one medical program across two medical schools with a unique approach to the student selection process so generalisability to other medical schools may be limited. Third, the study participants were accepted into medicine based on the scores of the tests used for selection, thus creating a restriction of range for each measure. If restricted range data is corrected for the purpose of correlational analysis then correlation parameters are under-estimated. This problem is common in medicine and organisational psychology selection studies which can only be overcome by experiments using random selection such as the lottery system employed in The Netherlands ( Visser et al. 2016).

Despite these limitations, this study importantly is the first to investigate the predictive validity of this unique combination of selection tools for entry into an undergraduate medical program in Australia. No other studies in Australia have examined the validity of MMIs and/or the PQA for entry into medicine to date. A large sample size of four student cohorts was utilised with a high (90%) participation rate providing consistency and generalizability across intake years. To-date, previous research has mainly relied on universal measures of student performance (such as GPA, examination marks) that are predominantly measures of cognition. This study utilised a broader range of outcome measures to reflect and capture - knowledge retention, analytical thinking, clinical skills, interpersonal skills and professional behaviour - all of which are important qualities for success as a medical student.

### Conclusion

The results of this study confirm the strength of prior academic results, namely ATAR scores, as a consistent and strong predictor of first year performance in academic outcomes but other selection tools do play a role in predicting one or more outcomes. It is generally acknowledged that the admissions process needs to encompass the full range of performance domains required of training doctors. Medical student selection needs to identify those candidates with sufficient potential for the levels of academic performance and professionalism required to succeed in a medical program in addition to being equitable and unbiased. Appraisal is required of each selection tool with respect to how they relate to available predictors of excellent patient care by our graduates and there is an ongoing responsibility for those involved to aim for optimal alignment of these parameters.

**Figure:**
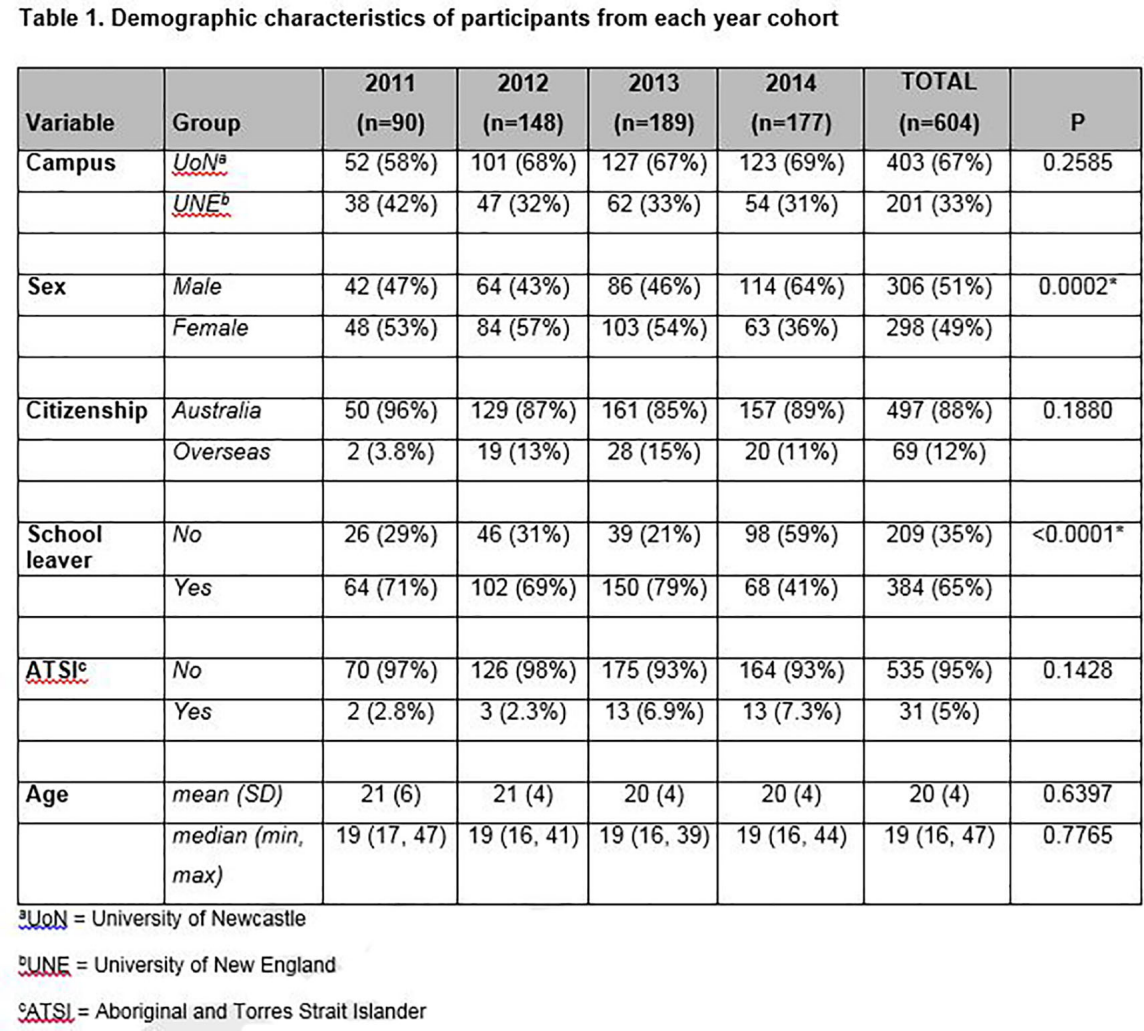


**Figure:**
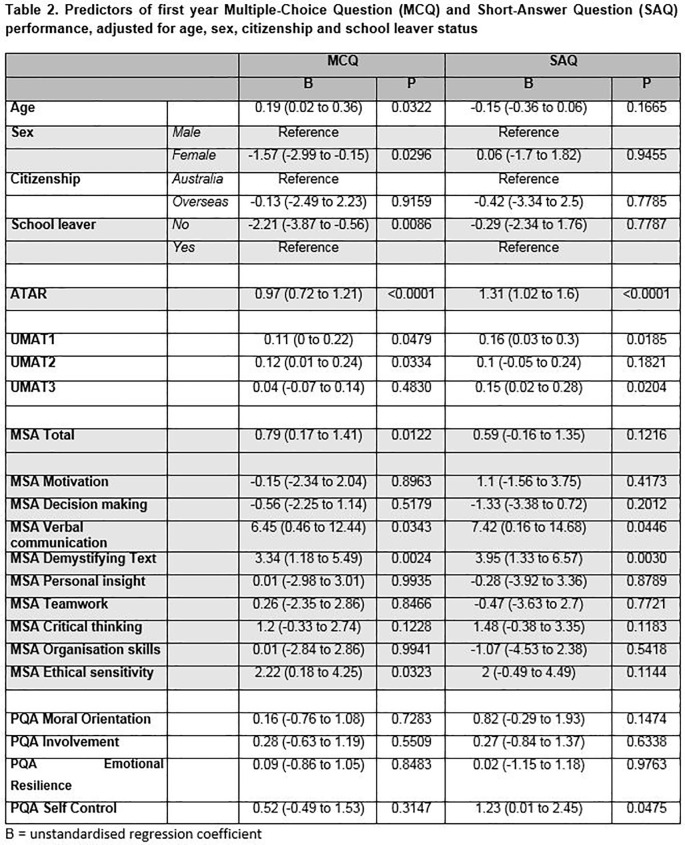


**Figure:**
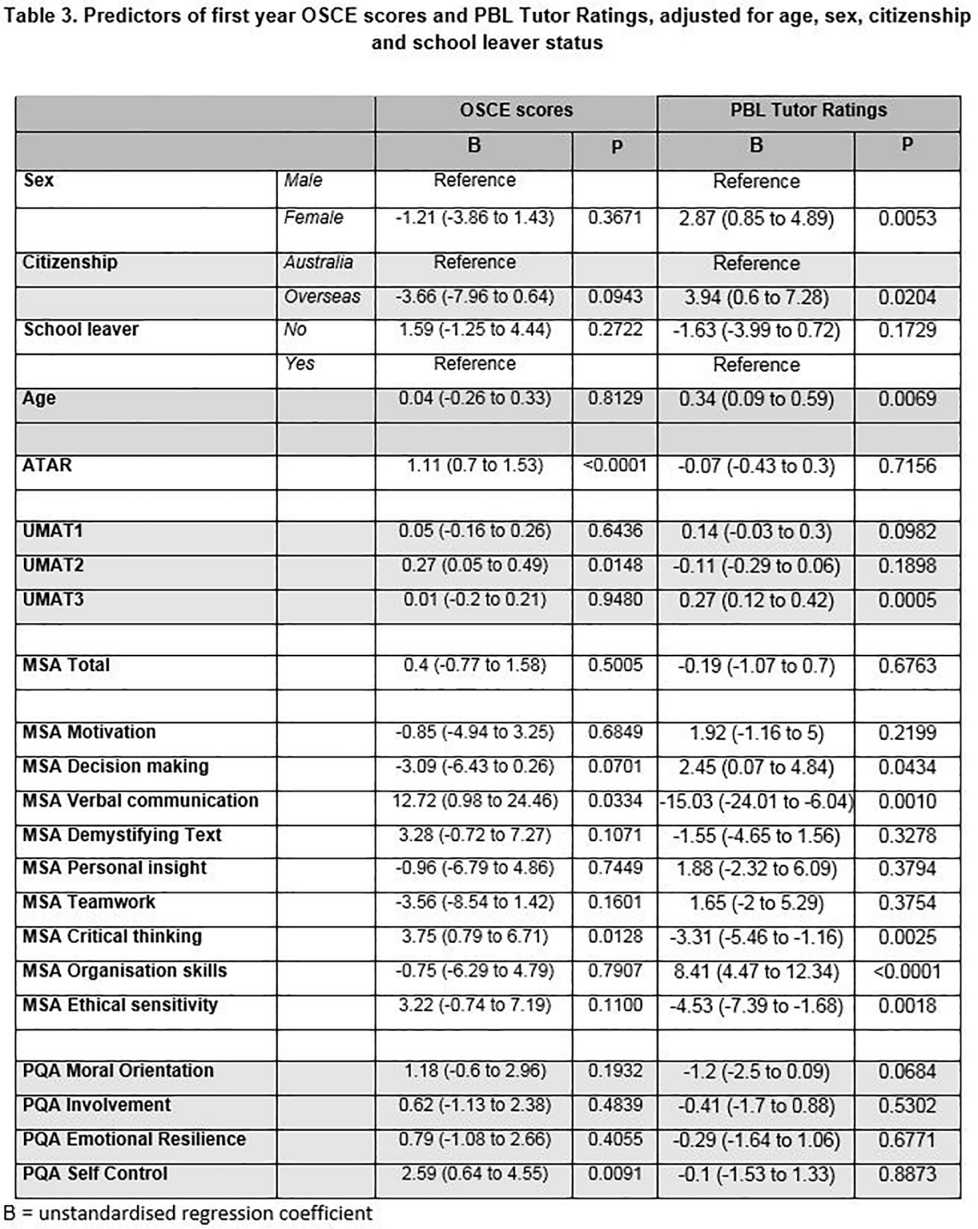


**Figure:**
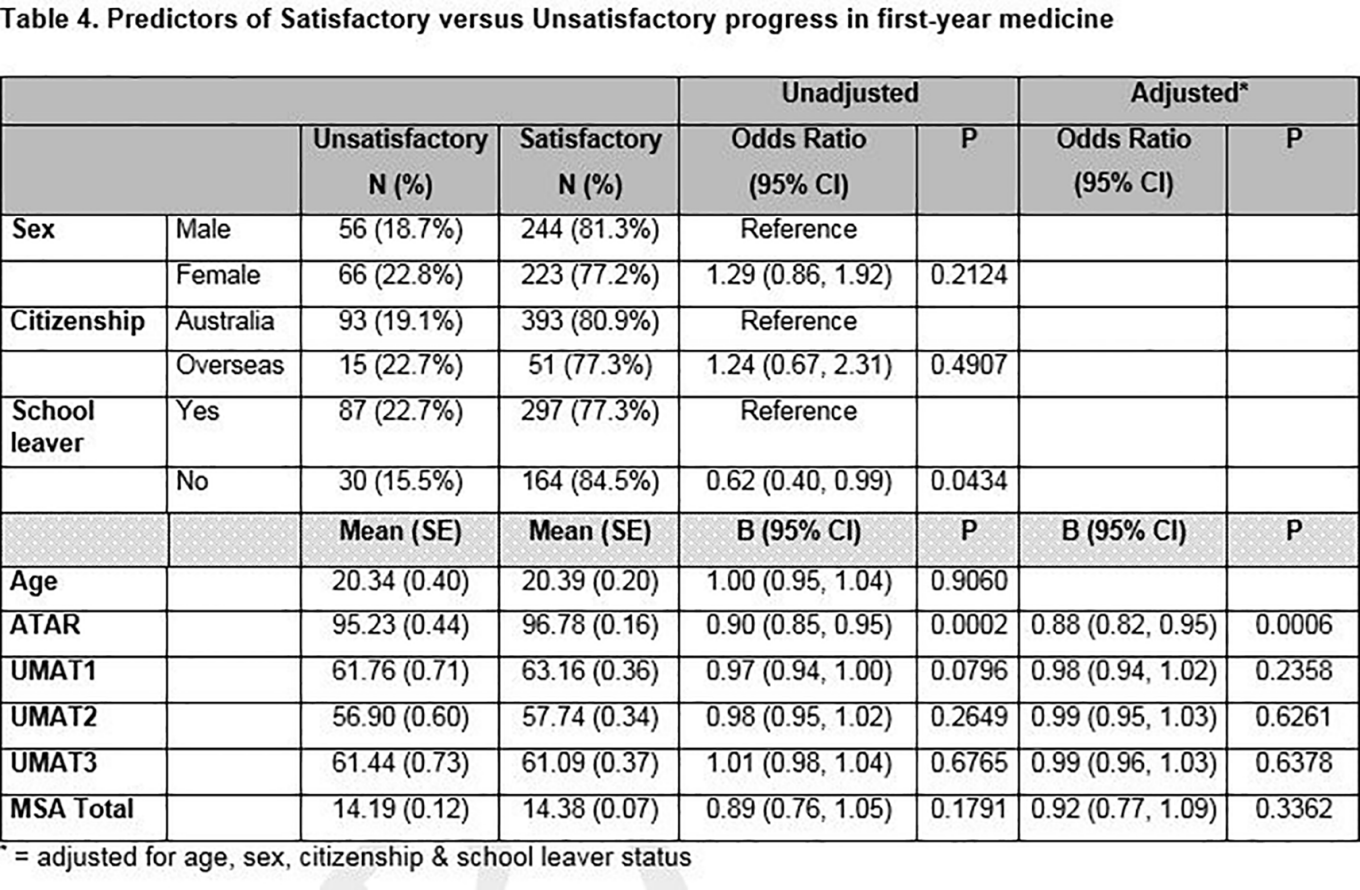


## Take Home Messages

• Medical schools utilise a range of selection tools to assess desirable cognitive and non-cognitive qualities in students entering medicine, but there is little research on how well these tools predict performance.
• Four selection tools (ATAR, UMAT, MMI & PQA) do predict one or more measures of academic performance in first year medicine.
• The overall combined predictive capacity of all tools in predicting first year performance is relatively low. This may be due to crude measures of student performance.
• Medical schools should continue to appraise their selection tools and consider assessment processes which more accurately capture performance across a range of performance domains.

## Notes On Contributors

MARITA LYNAGH, PhD, is Associate Professor in Health Behaviour Science at the University of Newcastle, Australia and chief investigator on this project evaluating the admissions process into medicine. Her other research interests include evaluating teaching programs for communication skills in medicine, incentives for health behaviour change and unmet needs of haematological cancer survivors.

BRIAN KELLY, BMed, PhD, is Professor in Psychiatry and Head of the School of Medicine and Public Health at the University of Newcastle, Australia. He has research expertise in rural mental health, palliative care and psycho-oncology, HIV infection/AIDS, ethical aspects of clinical practice and curriculum development in medical education.

GRAEME HORTON, BMed, is Senior Lecturer in Medical Education and Professional Development and Program Convenor for the Bachelor of Medicine at the University of Newcastle, Australia. His research interests include peer review of teaching and use of eportfolios in higher education. He is completing his PhD on the topic of climate change health impacts.

BEN WALKER, BMed, is Senior Lecturer in the School of Medicine and Public Health, University of Newcastle. His research interests include medical education and selection processes of admission into medicine.

DAVID POWIS, BMed, Phd, is Emeritus Professor in the School of Biomedical Sciences and Pharmacy and Conjoint Professor in the School of Psychology, University of Newcastle, Australia. He has a research focus on medical and health professional student selection processes and has worked with Miles Bore and Don Munro in developing and evaluating the Personal Qualities Assessment (PQA) tool.

MILES BORE, PhD, is Senior Lecturer and Deputy of Head of School in the School of Psychology, University of Newcastle, Australia. His research interests include personality, psychometrics and moral orientation. He is a member of the PQA research group.

DON MUNRO, PhD, is Associate Professor in the School of Psychology, University of Newcastle, Australia. His main research interests focus on personality, psychometrics and selection test construction. He is a member of the PQA research group.

IAN SYMONDS, BMed, PhD, is Professor of Reproductive Medicine and previously Head of School, School of Medicine & Public Health, University of Newcastle. He is currently Dean of Medicine at the University of Adelaide, Australia. He has research interests in obstetrics, gynaecology and medical education.

GRAHAM JONES, PhD, is Associate Professor and Convenor in Human Biology and Physiology, School of Science and Technology, University of New England, Australia. He has research interests in effects of antioxidant supplements, bioactivities on native Australia plants use by Aborigines and selection processes of entry into medicine.

AMANDA NAGLE, PhD, is Associate Professor and Year 1 Clinical Academic Co-ordinator, School of Rural Medicine at the University of New England, Australia. She is a behavioural scientist with research interests in lifestyle behaviour change at a population level, innovation diffusion, policy and systems change, and evaluation of new innovative medical education programs.

TIMOTHY REGAN, BPsych, is a Research Assistant in the School of Medicine and Public health, University of Newcastle, Australia.

PATRICK MCELDUFF, PhD, is Associate Professor in Biostatistics in the School of Medicine and Public Health, University of Newcastle, Australia.

MICHAEL DAVID, PhD is a Senior Lecturer in the School of Medicine and Public health, University of Newcastle, Australia. His research interests include cost-effectiveness analysis, medical education, meta-analysis and missing data.
