# Supplementary material for: Have we got the selection process right? The validity of selection tools for predicting academic performance in the first year of undergraduate medicine
Source: MedEdPublish (2016). 2017 Mar 9;6:42. [Version 1] doi: 10.15694/mep.2017.000042 (PMC10885238; doi:10.15694/mep.2017.000042)
Supplement: Peer review history

# Reviewer report: Reviewer response for version 1

**DOI:** 10.21956/mep.19450.r28639

**Published:** 2017-03-11

**Authors:** Peter Dieter

**Affiliations:** 1 AMSE

This review has been migrated. The reviewer awarded 4 stars out of 5 A paper on a project which is of high value for many medical schools worldwide: How can we select our students to 1) pass all necessary exams and 2) finally become a good doctor? Which tools can deliver a good prediction? Possibly we will never be satisfied with the results since the students are too young when they enter ths study (in my country with the age of 17-18 years) and we can not predict their development. Nevertheless, the paper describes a variety of tools which can be used. It might be very helpful for others working in this field although many countries do not use the tests and methods which are used in Australia..

Reviewer Expertise:

NA

No decision status is available

# Reviewer report: Reviewer response for version 1

**DOI:** 10.21956/mep.19450.r28642

**Published:** 2017-03-10

**Authors:** Linda Nield

**Affiliations:** 1 West Virginia University School of Medicine

This review has been migrated. The reviewer awarded 4 stars out of 5 This is well-written. It is helpful to read about the 4 tools implemented in the student selection process at these institutions. As my institution does not use MMIs, it would be interesting to read about the data results obtained specifically from the 2011 cohort when the structured interview panel was still being utilized.

Reviewer Expertise:

NA

No decision status is available

# Reviewer report: Reviewer response for version 1

**DOI:** 10.21956/mep.19450.r28640

**Published:** 2017-03-10

**Authors:** Trevor Gibbs

**Affiliations:** 1 AMEE

This review has been migrated. The reviewer awarded 4 stars out of 5 An interesting paper on an important subject. With the qualities required by graduating doctors increasing year upon year, it is hard to think that we will ever provide an answer to the questions raised in this paper; perhaps it is time to reflect that we will never find the one method but several acting complementary to each other; perhaps a time to apply an hierarchical list to differentiate between the must have, could have, nice to have qualities.I also believe the complexity of undergraduate education when combined with the intra-course development that occurs again means we may not find just one best method. Although I am unsure if this paper adds much to our present knowledge, it does reflect the the position of one Australian school and adds some useful references to those involved in the selection process

Reviewer Expertise:

NA

No decision status is available

# Reviewer report: Reviewer response for version 1

**DOI:** 10.21956/mep.19450.r28638

**Published:** 2017-03-10

**Authors:** Jennifer Cleland

**Affiliations:** 1 University of Aberdeen

This review has been migrated. The reviewer awarded 2 stars out of 5 this study determined the individual predictive validity of four different selection tools (UMAT subtests; ATAR scores, MSA, PQA) with academic performance outcomes in first-year undergraduate medical students, in one institution. This is a very typical type of study, but limited in its use as it does not explicitly acknowledge in its design that these tools are not used in isolation; rather they are combined and weighted in a selection process. The sequence of how these tools are used is clear but not their weighting, or how the tools are used in combination to reach a selection decision. There is little research into this area of medical education, although what evidence there is clearly identifies that diverse ways of weighting these tools (e.g., 50% for prior attainment, 30% UKCAT and 20% local assessment; or a hurdle model of “if over x, then through to the next stage”) will lead to different outcomes in terms of the population selected (e.g., Fernando et al., 2008; Tiffin et al., 2012; Patterson et al., 2016) The way tools are combined may add additional bias (ie additional to the individual bias of each individual tool) towards certain groups (e.g., Wright et al.,2010). Selection research needs to move on from looking at tools in isolation to research examining the impact of different weightings of admissions procedures on the equity and value of the overall selection process and widening access. ReferencesFernando N, Cleland JA, Greaves K, McKenzie H. Comparing the newly introduced UKCAT with the standardised admission selection process at a Scottish Medical School. Medical Teacher, 2009; 31: 1018-1023.Patterson F, Knight A, Dowell J, Nicholson S, Cleland JA. How effective are selection methods in medical education? A systematic review. Medical Education, 2016: 50: 36-60. Tiffin PA, Dowell JS, McLachlan JC. Widening access to UK medical education for under-represented socioeconomic groups: modelling the impact of the UKCAT in the 2009 cohort. BMJ 2012;344:e1805 Wright SR, Bradley PM. Has the UK Clinical Aptitude Test improved medical student selection? Med Educ. 2010 Nov;44(11):1069-76. doi: 10.1111/j.1365-2923.2010.03792.x.

Reviewer Expertise:

NA

No decision status is available

# Reviewer report: Reviewer response for version 1

**DOI:** 10.21956/mep.19450.r28641

**Published:** 2017-03-10

**Authors:** Richard Hays

**Affiliations:** 1 James Cook University

This review has been migrated. The reviewer awarded 4 stars out of 5 This paper addresses one of the perennial issues in medical education - can we select the 'right' people into primary medical programs from highly competitive fields? A common approach is to combine analysis of academic performance and personal qualities, the latter through a variety of measures. The results are not really new, but represent a comprehensive analysis that supports most other studies. Prior academic performance remains the most reliable predictor of at least short-term academic performance and other measures contribute a small amount. There are however other measures and longer term outcomes to consider. The really interesting finding here is that the predictive value of all measures combined is so small - 18%. Non-academic measures have strong face validity, and can be a method of engaging the community, but are they worth the effort and resource investment? This is not an easy question to answer. While this paper is about the admissions system at just one medical school, it contributes to that debate.

Reviewer Expertise:

NA

No decision status is available
